# Supplementary material for: Altered microRNA Transcriptome in Cultured Human Liver Cells upon Infection with Ebola Virus
Source: Int J Mol Sci. 2021 Apr 6;22(7):3792. doi: 10.3390/ijms22073792 (PMC8038836; doi:10.3390/ijms22073792)
Supplement: Supplementary file 1 [file ijms-22-03792-s001.zip › Supplementary_File/D_ KEGG_Analysis_Results/16-30nt_pathway_Makona-96h-Huh7_vs_Control-96h-Huh7_up.mature_mirna_targets/hsa_pathwayResult.html]

| PathwayID | Definition | OriginalWebSite | Fisher-Pvalue | SelectionCounts | SelectionSize | Count | Size | FDR | Enrichment\_Score | GeneRatio | Genes |
| --- | --- | --- | --- | --- | --- | --- | --- | --- | --- | --- | --- |
| hsa04070 | Phosphatidylinositol signaling system - Homo sapiens (human) | http://www.genome.jp/kegg-bin/show\_pathway?hsa04070+1606+4534+8394+23396+8867 | 1.182293e-03 | 5 | 64 | 99 | 7906 | 2.278080e-01 | 2.927275 | 0.078125 | DGKA//MTM1//PIP5K1A//PIP5K1C//SYNJ1 |
| hsa04145 | Phagosome - Homo sapiens (human) | http://www.genome.jp/kegg-bin/show\_pathway?hsa04145+8992+30835+10332+3115+7037+7057 | 1.368753e-03 | 6 | 64 | 152 | 7906 | 2.278080e-01 | 2.863675 | 0.093750 | ATP6V0E1//CD209//CLEC4M//HLA-DPB1//TFRC//THBS1 |
| hsa04668 | TNF signaling pathway - Homo sapiens (human) | http://www.genome.jp/kegg-bin/show\_pathway?hsa04668+9586+355+4049+1326+11035 | 2.046179e-03 | 5 | 64 | 112 | 7906 | 2.278080e-01 | 2.689056 | 0.078125 | CREB5//FAS//LTA//MAP3K8//RIPK3 |
| hsa00562 | Inositol phosphate metabolism - Homo sapiens (human) | http://www.genome.jp/kegg-bin/show\_pathway?hsa00562+4534+8394+23396+8867 | 2.940855e-03 | 4 | 64 | 74 | 7906 | 2.455614e-01 | 2.531526 | 0.062500 | MTM1//PIP5K1A//PIP5K1C//SYNJ1 |
| hsa04216 | Ferroptosis - Homo sapiens (human) | http://www.genome.jp/kegg-bin/show\_pathway?hsa04216+2730+8031+7037 | 4.038018e-03 | 3 | 64 | 40 | 7906 | 2.697396e-01 | 2.393832 | 0.046875 | GCLM//NCOA4//TFRC |
| hsa04940 | Type I diabetes mellitus - Homo sapiens (human) | http://www.genome.jp/kegg-bin/show\_pathway?hsa04940+355+3115+4049 | 4.957742e-03 | 3 | 64 | 43 | 7906 | 2.759810e-01 | 2.304716 | 0.046875 | FAS//HLA-DPB1//LTA |
| hsa05200 | Pathways in cancer - Homo sapiens (human) | http://www.genome.jp/kegg-bin/show\_pathway?hsa05200+578+8453+54567+355+4286+8648+8031+5915+6608+9618 | 9.460781e-03 | 10 | 64 | 529 | 7906 | 4.514144e-01 | 2.024073 | 0.156250 | BAK1//CUL2//DLL4//FAS//MITF//NCOA1//NCOA4//RARB//SMO//TRAF4 |
| hsa00561 | Glycerolipid metabolism - Homo sapiens (human) | http://www.genome.jp/kegg-bin/show\_pathway?hsa00561+56895+1606+346606 | 1.304368e-02 | 3 | 64 | 61 | 7906 | 5.445737e-01 | 1.884600 | 0.046875 | AGPAT4//DGKA//MOGAT3 |
| hsa04392 | Hippo signaling pathway - multiple species - Homo sapiens (human) | http://www.genome.jp/kegg-bin/show\_pathway?hsa04392+9770+83937 | 2.275958e-02 | 2 | 64 | 29 | 7906 | 7.652406e-01 | 1.642836 | 0.031250 | RASSF2//RASSF4 |
| hsa04721 | Synaptic vesicle cycle - Homo sapiens (human) | http://www.genome.jp/kegg-bin/show\_pathway?hsa04721+8992+8218+6804 | 2.503112e-02 | 3 | 64 | 78 | 7906 | 7.652406e-01 | 1.601520 | 0.046875 | ATP6V0E1//CLTCL1//STX1A |
| hsa05162 | Measles - Homo sapiens (human) | http://www.genome.jp/kegg-bin/show\_pathway?hsa05162+578+30835+10332+355 | 2.520254e-02 | 4 | 64 | 138 | 7906 | 7.652406e-01 | 1.598556 | 0.062500 | BAK1//CD209//CLEC4M//FAS |
| hsa04072 | Phospholipase D signaling pathway - Homo sapiens (human) | http://www.genome.jp/kegg-bin/show\_pathway?hsa04072+56895+1606+8394+23396 | 3.151229e-02 | 4 | 64 | 148 | 7906 | 8.293672e-01 | 1.501520 | 0.062500 | AGPAT4//DGKA//PIP5K1A//PIP5K1C |
| hsa05216 | Thyroid cancer - Homo sapiens (human) | http://www.genome.jp/kegg-bin/show\_pathway?hsa05216+578+8031 | 3.582755e-02 | 2 | 64 | 37 | 7906 | 8.293672e-01 | 1.445783 | 0.031250 | BAK1//NCOA4 |
| hsa05330 | Allograft rejection - Homo sapiens (human) | http://www.genome.jp/kegg-bin/show\_pathway?hsa05330+355+3115 | 3.762421e-02 | 2 | 64 | 38 | 7906 | 8.293672e-01 | 1.424533 | 0.031250 | FAS//HLA-DPB1 |
| hsa05222 | Small cell lung cancer - Homo sapiens (human) | http://www.genome.jp/kegg-bin/show\_pathway?hsa05222+578+5915+9618 | 3.817351e-02 | 3 | 64 | 92 | 7906 | 8.293672e-01 | 1.418238 | 0.046875 | BAK1//RARB//TRAF4 |
| hsa05332 | Graft-versus-host disease - Homo sapiens (human) | http://www.genome.jp/kegg-bin/show\_pathway?hsa05332+355+3115 | 4.321570e-02 | 2 | 64 | 41 | 7906 | 8.293672e-01 | 1.364358 | 0.031250 | FAS//HLA-DPB1 |
| hsa00564 | Glycerophospholipid metabolism - Homo sapiens (human) | http://www.genome.jp/kegg-bin/show\_pathway?hsa00564+56895+1606+55224 | 4.357285e-02 | 3 | 64 | 97 | 7906 | 8.293672e-01 | 1.360784 | 0.046875 | AGPAT4//DGKA//ETNK2 |
| hsa05231 | Choline metabolism in cancer - Homo sapiens (human) | http://www.genome.jp/kegg-bin/show\_pathway?hsa05231+1606+8394+23396 | 4.469643e-02 | 3 | 64 | 98 | 7906 | 8.293672e-01 | 1.349727 | 0.046875 | DGKA//PIP5K1A//PIP5K1C |
